# Variation in Nutrient Composition of Seafood from North West Africa: Implications for Food and Nutrition Security

**DOI:** 10.3390/foods9101516

**Published:** 2020-10-21

**Authors:** Inger Aakre, Annbjørg Bøkevoll, Jamal Chaira, Fatima Zohra Bouthir, Sylvia Frantzen, Anette Kausland, Marian Kjellevold

**Affiliations:** 1 Institute of Marine Research, 5817 Bergen, Norway; Annbjorg.Bokevoll@hi.no (A.B.); Sylvia.Frantzen@hi.no (S.F.); anette.kausland@gmail.com (A.K.); marian.kjellevold@hi.no (M.K.); 2 National Institute for Fisheries Research (INRH), Casablanca 20030, Morocco; jamal_chaira@yahoo.fr (J.C.); fzbouthir@gmail.com (F.Z.B.)

**Keywords:** fish, nutrient composition, recommended nutrient intakes, micronutrients, food and nutrition security, Africa, food composition data

## Abstract

Fish and seafood may play an important role for nutrition and food security as they contain essential vitamins, minerals, and essential fatty acids. The aim of this study was to describe the nutrient composition, including fatty acids, amino acids, vitamins, and minerals, in commonly consumed fish species (fillet- and whole fish samples) sampled off the Northwest African coast. Furthermore, we assessed the species’ contributions to the recommended nutrient intake (RNI) values from the World Health Organization (WHO). Samples of commercially important fish species (*Sardina pilchardus, Engraulis encrasicolus, Trachurus trachurus, Pagellus acarne*) were collected using trawling on the R/V Dr. Fridtjof Nansen in May 2017 and analyzed for nutrients at the Institute of Marine Research as individual and composite samples. All the analyzed fish species were good dietary sources of several vitamins and minerals and whole fish were substantially more nutrient dense than fillet samples, especially with regard to vitamin A, iodine, zinc, calcium, and iron. Including 100 g of sardine or anchovy (whole fish) in the diet, would contribute substantially to the RNI for vitamin B_12_, vitamin D and vitamin A, EPA and DHA as well as the minerals iodine, zinc, and calcium. This study shows that fish consumed with skin, bone, and viscera may be very nutrient dense and important for local food and nutrition security.

## 1. Introduction

Fish and seafood are important parts of the diet for people in many countries in the Northwest African region [1,2]. Seafood plays a central role for food and nutrition security [3], as it contributes with several essential nutrients, which are important for human health [4,5]. Fish are a high quality protein food and include essential amino acids [5], and fatty acids such as the long-chain *n*-3 polyunsaturated fatty acids (*n*-3 PUFAs), of which eicosapentaenoic acid (EPA, 20:5*n*-3) and docosahexaenoic acid (DHA, 22:6*n*-3) are considered beneficial for human health [6,7,8]. Seafood is also a good source of micronutrients including iodine, selenium, vitamin B_12_, and vitamin D [5,9,10]. Small fish eaten whole with bones may also contribute with significant amounts of calcium, zinc, iron, and vitamin A in the human diet [11]. Nutrient composition of fish and seafood may exhibit significant variation within and among species, depending on tissue type, habitat, region, and season [12,13,14].

The marine areas off the coast of Northwest Africa are situated in the southern region of the Canary current ecosystem, where the upwelling of nutrient-rich deep water leads to a high production of pelagic fish species such as sardines, anchovies, and mackerel [15]. According to the Food and Agriculture Organization of the United Nations (FAO), the capture production in 2016 of the most abundant fish species off the Northwest African coast, European pilchard (*Sardina pichardus*), was estimated to be >900,000 tons [16]. The capture production of European anchovy (*Engraulis encrasicolus*) was ≈26,000 tons and horse mackerel totaled ≈39,000 tons [16].

There is currently little data on the nutritional composition of seafood from the North West African region. The food composition table in West Africa shows values for macronutrients and some minerals for some common species, such as anchovy, sardines, and mackerel [17]. However, most of these values are calculated, borrowed, and/or estimated. Very few analytical data exist from the area, but some published values have been found for a few nutrients [18,19,20,21]. Food composition data are foundational to nutrition and public health-related investigations, dietary evaluations, and for the development of effective food- and nutrition related policies [22]. Therefore, it is critical that high-quality and available food composition data are published on a country-specific basis [23,24].

Here we present the results of analyses of a wide range of nutrients including fatty acids, amino acids, vitamins, and minerals in commonly consumed fish species (both whole fish samples and fillet), sampled from the North West African marine ecosystem (Tangier to Cap Bojador) on the research vessel *R/V* Dr. Fridtjof Nansen in May 2017. We assess the different species’ contributions to recommended nutrient intake values from the WHO and discuss the implication of our findings for regional food and nutrition security.

## 2. Materials and Methods

This paper uses data collected through the scientific surveys with the R/V Dr. Fridtjof Nansen as part of the collaboration between the EAF-Nansen Programme and National Institute for Fisheries Research (INRH) Morocco. The EAF-Nansen Programme is a partnership between the Food and Agriculture Organization of the United Nations (FAO), the Norwegian Agency for Development Cooperation (Norad), and the Institute of Marine Research (IMR), Norway for sustainable management of the fisheries of partner countries [25,26].

### 2.1. Sampling and Sample Preparation

Fish samples were collected during an ecosystem survey with R/V Dr. Fridtjof Nansen from Tangier to Cap-Bojador from 8 to 26 May 2017. Pelagic (MultiPelt 624) and demersal trawls (Gisund Super bottom trawl) were towed throughout the survey. The catch was categorized, sorted, and identified to species by taxonomists. The fish species selected for sampling were among the most common species in the area and species commonly consumed by humans. Fish were sampled for analysis (e.g., whole fish or fillet) based on how each species is prepared by local consumers, according to local scientists.

Sample preparation was completed in the laboratories on board the *R/V* Dr. Fridtjof Nansen. Species tissue and sample type were recorded and are described in Table 1. Fish samples were prepared according to predeveloped protocols, one for “small” fish (<25 cm) and one for “large” fish (>25 cm). Small fish species included sardines (*Sardina pilchardus*) and anchovies (*Engraulis encrasicolus*) and were analyzed as composite samples of 25 whole fish and 25 fillets with skin and bone (whole fish with head and viscera removed). Detailed information about sampling procedures and protocols are described elsewhere [27]. From each sampling site, three composite samples of each tissue type were made. The larger fish species Atlantic horse mackerel (*Trachurus trachurus*) and axillary seabream (*Pagellus acarne*) were analyzed as fillet samples, either individually or as composite samples depending on the laboratory analyses. Large fish were analyzed as composite samples for selected vitamins with fillets of five individuals included in each composite sample. For the proximal values and trace elements, fillet samples from individual fish were analyzed.

All samples were homogenized using a food processor (Braun Multiquick 7 K3000 Kronberg im Taunus, Germany). For each sample, a wet sample and a freeze-dried sample were prepared. Freeze-drying was performed using a Labconco Freezone instrument (18 L mod. 7750306, Kansas City, KS, USA). Frozen samples (minimum 12 h at −20 °C) were freeze dried for at least 72 h until a constant mass was achieved, and all moisture was removed. Both wet and freeze-dried samples were vacuum sealed and stored in insulated boxes at −20 °C. The samples were shipped by air cargo to Bergen, Norway, where the samples were stored at IMR at −80 °C pending laboratory analyses.

### 2.2. Analytical Methods

The determination of nutrients in the fish samples were done at the laboratories at IMR, Norway. All the analyses were performed by accredited methods according to ISO 17025:2005. Energy and iron analyses are validated methods, but not accredited. The accuracy and precision in addition to the measurement uncertainty of each specific method has been tested regularly by analyzing certified reference materials and by participation in national and international proficiency tests. All values of certified reference materials (CRM) were within the accepted area of the analyses. Self-produced internal control material or reference materials was used to check daily control of the method. All the samples were homogenized prior to analyses. For total fat, total protein, amino acids, and minerals, freeze-dried and pulverized sample material was used. Analyses for vitamins and fatty acids were performed on wet sample material. An overview of the analytical methods used for each nutrient, their validated measurement range (including limits of quantification (LOQ)), measurement uncertainties, results for CRM and internal control materials are described elsewhere [27].

### 2.3. Determination of Crude Fat and Protein, Ash, Energy, Fatty Acids, Amino Acids, Vitamins, and Minerals

Fat (crude fat) was extracted with ethyl acetate/isopropanol (70/30) and filtered before the solvent was evaporated and the fat residue was weighed. The method is based on a Norwegian Standard, NS 9402 [28]. Protein (crude protein) was determined by burning the material in pure oxygen gas in a combustion tube (Leco FP 628, Leco Corporation, Saint Joseph, MI, USA) at 950 °C. Nitrogen (N) was detected with a thermal conductivity detector (TCD Leco Corporation, Saint Joseph, MI, USA) and the content of N was calculated from an estimated average of 16% N per 100 g protein. The following formula was used: g N/100 g × 6.25 = g protein/100 g, in accordance with the method accredited by the Association of Official Agricultural Chemists (AOAC) [29].

Ash is defined as the inorganic residue obtained after removal of moisture and organic matter by heat treatment. Analysis of ash content was performed according to NMKL Method 23.3 [30] and with use of a muffle oven (Thermolyne F 30,430 CM, Thermo Fisher Scientific, Waltham, MA, USA).

Energy was measured using an automatic isoperibol calorimeter (Parr Calorimeter 6400–Moline, Illinois, IL, USA). For determination of fatty acids (FA), lipids from the samples were extracted according to Folch et al. [31] and FA composition of total lipids was analyzed as previously described [32,33] by use of the software Chromeleon^®^ version 7.1 (Thermo Scientific Dionex, Waltham, MA, USA) connected to the gas liquid chromatograph (GLC) with a Flame Ionization Detector (FID) (Perkin Elmer Auto System XL2000 gas chromatograph, Perkin Elmer, Waltham, MA, USA). Amount of FA per gram sample was calculated using 19:0 methyl-ester as internal standard.

Amino acids were analyzed using UPLC (reverse phase) and UV detection (Waters Acquity UPLC System, Waters, Milford, MA, USA). Quantification was determined using internal and external standard curves (Waters, AccQ-Tag™ Method 715001320, REV D [34]).

Tryptophan was analyzed by HPLC (reverse phase) using a UV detector (Agilent 1290 Infinity system, Agilent Technologies, PDA, Santa Clara, CA, USA). Tryptophan content was calculated by external calibration (standard curve). The method is based on a previously described method [35,36] with modifications.

Vitamin A_1_ (sum all trans retinol and 13-, 11-, 9 cis retinol) was determined by HPLC (normal phase) using a Photo Diode Array detector (HPLC 1260 system Agilent Technologies, PDA, Santa Clara, CA, USA). The retinol content was calculated by external calibration (standard curve) [37]. Vitamin E (α-, β-, γ-, δ-tokoferol and α-, β-, γ-, δ-tokotrienol) was determined by HPLC (normal phase) using a fluorescence detector (HPLC UltiMate3000 system, Thermo Fisher Scientific, Waltham, MA, USA). The vitamin E content was calculated by external calibration (standard curve) [38]. Vitamin D_3_ was cleaned up on preparative HPLC (normal phase) and determined by HPLC column (reverse phase) and UV detector (HPLC LaChrom Merck HITACHI system, Tokyo, Japan). The content of vitamin D_3_ was calculated using internal standard (vitamin D_2_) [39].

Vitamin B_1_ (Thiamin) was released from the sample by acid extraction, hydrolysis, and enzyme treatment. Further post-column derivation (reverse phase) of thiamine to thiochrome, prior to detection by a fluorescence detector (Agilent 1100 HPLC system, Agilent Technologies, PDA, Santa Clara, CA, USA). Vitamin B_1_ content was calculated by external calibration (standard curve) [40]. Vitamin B_2_ (Riboflavin) was released from the sample by acid extraction, hydrolysis, and enzyme treatment and determined by HPLC (reverse phase) using a fluorescence detector (Agilent 1100 HPLC system, Agilent Technologies, PDA, Santa Clara, CA, USA). The content was calculated by external calibration (standard curve) [41]. Vitamin B_6_ (pyridoxsine, pyridoxsal, and pyridoxsamin) was determined by HPLC (reverse phase) using a fluorescence detector (Agilent 1290 Infinity HPLC system, Agilent Technologies, PDA, Santa Clara, CA, USA). Vitamin B_6_ content was calculated by external calibration (standard curve) [42]. Vitamin B_3_ (Niacin) was released from the sample by extraction (autoclaving in sulfuric acid) and mixed with growth medium, added to the microorganism *Lactobacillus plantarum* (ATCC 8014), and incubated at 37 °C for 22 h. The vitamin content was calculated by comparing the growth of the organism in the unknown samples with the growth of the organism in known standard concentrations with turbidimetric reading (Optical Density, OD, v/575 nm) [43]. Vitamin B_9_ (Folic acid) was released from the sample by extraction (autoclaving in acetate buffer) and mixed with growth medium, added the microorganism *Lactobacillus rhamnosus* (ATCC 7469), and incubated at 37 °C for 20 h. The vitamin content was calculated by comparing the growth of the organism in the unknown samples with the growth of the organism in known standard concentrations, using turbidimetric reading (Optical Density, OD, v/575 nm) (method based on the Swedish Nestlé AB’s microbiological determination of folic acid in food, method nr. 71 C-2). Vitamin B_12_ (Cobalamin) was released from the sample by extraction (autoclaving in acetate buffer) and mixed with growth medium, the microorganism *Lactobacillus delbruecki* (ATCC 4797) added, and incubated at 37 °C for 22 h. The vitamin content was calculated by comparing the growth of the organism in the unknown samples with the growth of the organism in known standard concentrations, using turbidimetric reading (Optical Density, OD, v/575 nm) [43].

The concentration of minerals iodine (I), (selenium (Se), zinc (Zn), iron (Fe), calcium (Ca), potassium (K), magnesium (Mg), phosphorous (P), and sodium (Na) were determined by Inductively Coupled Plasma-Mass Spectrometry (iCapQ ICP-MS, ThermoFisher Scientific, Waltham, MA, USA) equipped with an autosampler (FAST SC-4Q DX, Elemental Scientific, Omaha, NE, USA) after wet digestion in a microwave oven (UltraWave or UltraClave, Milestone, Sorisole, Italy) [44]. The concentration of these elements was determined using an external standard curve in addition to an internal standard. Three slightly different methods were applied, (1) for Ca, Na, K, Mg, and P using scandium (Sc) as internal standard, (2) for Zn and Se using rhodium (Rh) as an internal standard, and (3) for I tellur (Te) was used as internal standard. For the determination of iodine, the sample preparation is a basic extraction with tetramethylammonium hydroxide (TMAH) before ICP-MS analysis [44]. Further detail regarding the analytical methods has been published elsewhere [27].

### 2.4. Data Management

All analytical values were exported from Laboratory Information Management Systems (LIMS) (Labware, Wilmington, DE, USA) into excel and quality assured. Data management was done in EXCEL and IBM SPSS version 22 (IBM Corp., Armonk, NY, USA). Single values below the LOQ were given as below the respective limit for each analyte. When calculating mean and variance, values below the LOQ were entered into the dataset as the respective LOQ divided by two. For vitamin B_1_ over 15% of the values for anchovy were below the LOQ, thus these numbers should be used with caution, as the uncertainties are large.

One hundred grams of the different fish species were used to estimate how much one portion of the respective fish contributed to the recommended intake for adult women using the Recommended Nutrient Intakes (RNI) from the WHO and FAO [45]. For EPA and DHA, the Dietary Reference Values from the European Food Safety Authority (EFSA) were used [46].

## 3. Results

All of the fish sampled were commonly distributed species in the Atlantic Ocean, including the coastal areas off Northwestern Africa (https://www.fishbase.in/home.htm). For each species of fish, the weight, length, and habitat are described in Table 2. Anchovy was the smallest species with an average length of 13 cm and a maximum of 14 cm, while Atlantic horse mackerel was the largest species sampled, with an average length of 31 cm.

Table 3 describes the energy and water content, ash, total fat, and total protein in the seafood samples. Sardine and anchovy had the highest fat contents, with mean values of 7.8 and 4.3 g/100 g in whole fish of the two species, respectively, and similar values in fillet with skin and bone. The remaining samples had fat contents below 1 g/100 g. Sardine and anchovy were also the two most energy dense fish species, with mean energy contents of 161 and 141 Kcal/100 g in whole fish of the two species, respectively.

The contents of selected fatty acids in the seafood sampled are given in Table 4. As seen from the table, sardines and anchovies which had the highest contents of fat, also had the highest contents of the essential *n*-3 fatty acids EPA and DHA, with average values in whole fish for EPA and DHA, respectively, of 1.2 and 0.87 g/100 g in sardines and 0.54 and 1.0 g/100 g in anchovy.

The contents of essential amino acids and taurine in the various seafood samples are described in Table 5. All species sampled had relatively high contents of total protein and amino acids. The fillet samples had slightly higher contents of amino acids than the whole fish samples.

Analytical values for selected vitamins are shown in Table 6. The vitamin D content varied widely across species and was highest in horse mackerel fillets (mean = 28.4 µg/100 g) and lowest in anchovy fillet (1 µg/100 g). Vitamin A_1_ content was also highly variable among species, being particularly high in the whole fish samples of sardines and anchovies with mean values 115 and 125 µg/100 g, respectively. Fillet (with skin and bone) of anchovy had the third highest Vitamin A_1_ content, with a mean of 7.0 µg/100 g. For vitamin E, both tocopherols and tocotrienols, including their α-, β-, γ-, and δ-forms were analyzed, but only α-tocopherol is shown in the table. For the tocotrienols, all analyzed values were below the LOQ (8 µg/100 g) for all species, except from one of the composite samples of axillary seabream where the δ-form had a mean of 23.8 µg/100 g (data not shown). For the tocopherols, the β-form was below LOQ (4 µg/100 g) for most species, but sardine and anchovy had values slightly above the LOQ. For the γ-form all values were below LOQ and for the δ-form all species were below the LOQ (data not shown).

The contents of the different B-vitamins in the analyzed seafood samples are shown in Table 7. Niacin, B_12_, and folic acid were the only B-vitamins with any significant amounts in the species sampled, while the levels of thiamin, riboflavin, and pyridoxine were low in all species sampled. In sardines and anchovies, folic acid content was considerably higher in whole fish compared to fillets with skin and bone.

The contents of selected minerals and trace elements in the analyzed seafood samples are given in Table 8. All elements except potassium were higher in the samples of whole fish compared with the samples of fillet for sardine and anchovy. Whole-bodied sardines were the most mineral rich species and also had the highest observed content of iodine, selenium, calcium, zinc, iron, and phosphorous.

**Figure 1:**
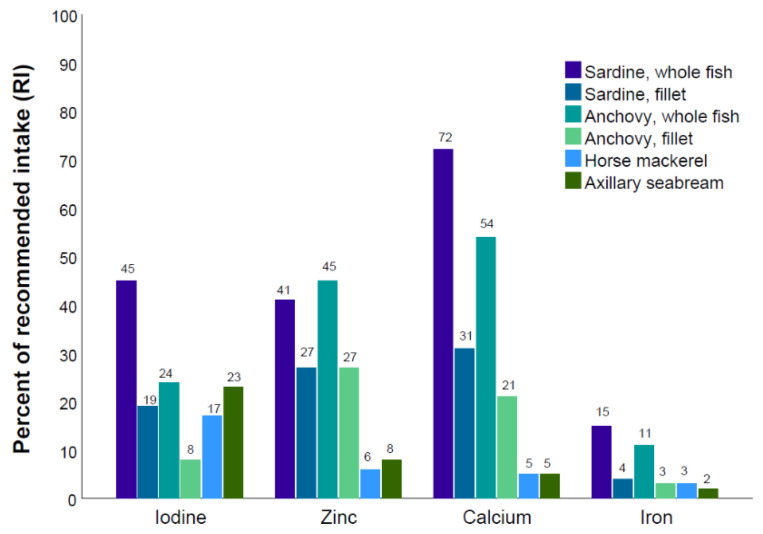
Percent of recommended intake of selected minerals and trace elements for 100 g of sardine (whole fish and fillet), anchovy (whole fish and fillet), horse mackerel, and axillary seabream. Recommended Nutrient Intake (RNI) values from WHO are used (WHO, 1998).

**Figure 2:**
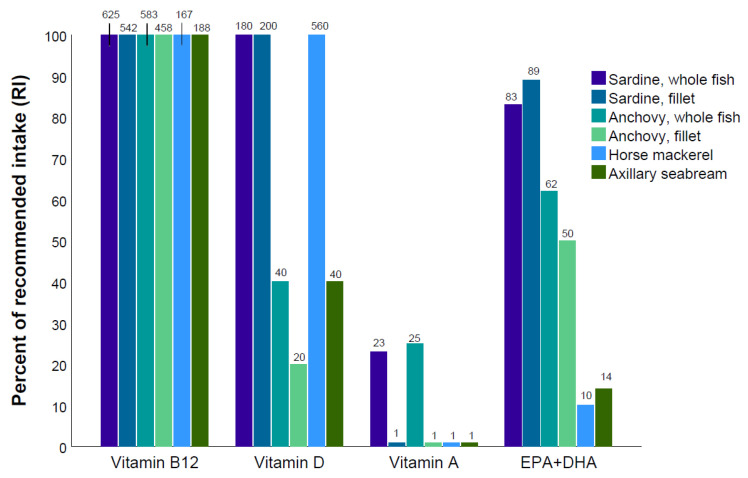
Percent of recommended intake of selected vitamins and EPA + DHA of 100 g of sardine (whole fish and fillet), anchovy (whole fish and fillet), horse mackerel, and axillary seabream sampled from Northwestern African marine ecosystems, May 2017. Recommended Nutrient Intake (RNI) values from WHO are used (WHO, 1998) with the exception of EPA + DHA where the Dietary Reference Values (DRV) from EFSA are used (EFSA, 2010).

Figure 1 and Figure 2 illustrate the contribution of 100 g of the different fish species in percentage of the daily recommended nutrient intake (RNI) for adult women. As seen from Figure 1, sardine and anchovy had the highest contribution to the RNI for iodine, zinc, calcium, and iron. Whole fish were substantially more nutrient dense than fillet from the same species. Horse mackerel and axillary seabream had lower contents of the selected micronutrients than sardine and anchovy. As seen in Figure 2, all the species met the daily recommendation for vitamin B_12_. Sardine and horse mackerel provided more than 100% of the recommended nutrient intake for vitamin D. Sardine and anchovy were also good sources of vitamin A, but only as whole fish and to a lesser degree as fillets. Both sardines and anchovies were good dietary sources of EPA and DHA, as both whole fish and fillets with skin and bone and provided more than 50% of the RNI. Horse mackerel and axillary seabream, which were the only species where skin- and boneless fillets were analyzed, did not contribute much with regard to EPA and DHA, only 10 and 14% of RNI, respectively.

## 4. Discussion

This paper presents analytical values for a wide array of nutrients in commonly consumed fish species caught off the Northwest coast of Africa. All the fish species included in this paper are significant dietary sources of micronutrients, protein, and fatty acids. The most nutrient-rich fish species were sardines and anchovies, and whole fish were substantially more nutrient dense than fillets.

Fish is an important part of the diet in many of the Northwestern African countries and contributes >20% of animal protein supply in countries such as Morocco, Senegal, and Gambia [47]. All the fish species included in this paper were categorized as lean or intermediate fatty fish, with fat contents varying from 0.6 to 7.8 g/100 g [48]. Therefore, energy content was quite low compared to fatty fish species such as herring and salmon [49]. However, as fish is not a staple food, the energy content is secondary to the contents of micronutrients and essential fatty acids, which are the nutrients that may be scarce in plant-based diets or in areas with high levels of food insecurity [50]. Adequate intake of nutrients such as iron, zinc, iodine, and vitamin B_12_ may be difficult, or even impossible to obtain from plant source foods alone [51], and fish is therefore an important addition to the diet.

In this study, sardines and anchovies had the highest contents of EPA and DHA, where 100 g anchovy and sardine fillets contributed with 89% and 50% of the recommended nutrient intake for adult women, respectively. The levels of EPA and DHA in sardines (both whole fish and fillet) were higher compared to levels found in muscle tissue of *Sardinella maderensis* and *S. aurita* in a study along the coast of Senegal and Mauritania [52]. Sardines and anchovies were also the most mineral and trace element rich fish species. Moreover, for sardine and anchovy, the whole fish samples had higher concentrations of iodine, selenium, calcium, magnesium, zinc, iron, and phosphorus compared to samples of fillet with skin and bone. The differences may be caused by the presence or absence of bones, skin, and viscera in the samples, as well as inclusion of the liver [53]. Fish bones are naturally rich in minerals, particularly calcium and phosphorous [54,55,56], which may explain the higher mineral content in the whole fish samples compared with fillet. Likewise, a study of metals in skin, liver, and muscle of *Lethrinus lentjan* found that liver and skin were high in zinc compared to muscle tissue [57]. Furthermore, a study of several species caught along the coast of Mauritania found similar results, where zinc concentrations were substantially higher in liver and gills than in muscle tissue [58]. The whole fish samples of sardines and anchovies were also a significant source of iron, which may have been due to the inclusion of head and viscera. Iron is mostly found in the liver, other entrails, and blood, which may explain the higher content of iron in whole fish compared to fillets. Other investigators as well, have reported that whole fish may contribute with dietary iron [59], and even though the study included small indigenous species from Bangladesh we believe these findings are of relevance in other contexts. Whole fish samples had much higher content of vitamin A compared to fillets. Whole fish as an important dietary source of vitamin A has been reported by others and is likely caused by high concentrations of Vitamin A in the eye and liver [11,59,60]. To prepare and consume whole fish is customary in many parts of the Northwest African region and may be important for food and nutrition security as inclusion of bones, skin, head, and viscera has been reported to significantly increase the mineral contents of these food sources [61,62].

All fish species were very high in vitamin B_12_ and 100 g of fish contributed >100% of the RNI. Sardines and horse mackerel were the fish species with the highest contents of vitamin D. The fatty fish species are, in general, expected to have a higher content of vitamin D compared to lean species, however the findings in this study are not in accordance with this assumption. Horse mackerel, with a fat content below 1 g/100 g had the highest content of vitamin D. Vitamin D levels independent of fat content in several species of fish have likewise been reported by other investigators [63].

We found that including 100 g of fish, and especially whole fish, would contribute significantly to the RNI for several nutrients (Figure 1 and Figure 2). The prevalence of undernourishment, such as stunting and wasting in children, micronutrient deficiencies but also overweight and non-communicable diseases are significant public health problems in parts of the Northwest African region [64]. In order to combat undernourishment and micronutrient deficiencies, there is an increasing focus on the role of nutrition-sensitive interventions, including food-based approaches. Fish, which contains bioavailable micronutrients as well as essential fatty acids and amino acids, may be an important component in order to achieve sustainable improvements in human nutrition [11,50,65]. Additionally, fish has a central role in a healthy diet and may contribute in the prevention of diet-related diseases [66].

The West African Food Composition Table (WA-FCT) [17] encloses compiled data for selected nutrients in raw sardines (tissue type unknown), anchovies (fillets), and mackerel (fillets). The levels of vitamin B_12_ were higher in the three species analyzed in this investigation, whereas the levels of vitamins D and A were higher in the WA-FCT for anchovies and mackerel, respectively. For sardines, Ca, Fe, P, and Zn were higher in this investigation and Mg and K were higher in the WA-FCT. The fillet samples of anchovies had similar values for K, Zn, and Mg, whereas Ca and P were higher, and Fe was lower in our data compared with fillet samples of anchovies in the WA-FCT. Similar values were observed in mackerel for Fe, Mg, Zn, and P, while our data showed higher levels of Ca and K [57]. However, the compiled data from the WA-FCT for the current fish species lack local and analytical data. Most values are borrowed from US or Danish FCTs or papers published from other geographical locations. Therefore, the results from this investigation should be considered an important update as they provide high quality data using accredited laboratory analytical methods.

Accurate and reliable food composition data is the foundation for research regarding nutrition, public health, and food security. Moreover, nutritional data are important in order to estimate nutrient intakes, to assess nutrient requirements, and to develop effective and accurate nutrition and food security policies [23,67]. In this study the analytical samples of fish consisted of a minimum of 25 individuals from different sampling positions. This is in accordance with most standards for food composition data, where at least 10 units are used [22,68], and with the U.S. standards for nutrition labeling which requires 12 units [22]. However, the nutrient content may vary between different maturation stages of the fish, by areas and seasons and therefore samples used in this study may have some notable limitations.

In this study we did not report contaminant concentration in the sampled fish. Fish and seafood are one of the major routes of arsenic, cadmium, mercury, and lead exposure in humans [69]. Although fish eaten whole may be an important source of essential nutrients, it may also be a potential source of contaminants, as studies have reported accumulation of heavy metals in liver and gills [70,71]. Moreover, the fish included in our study was raw samples. Several factors may influence the nutrient and contaminant levels during processing, which may influence on the nutrient contribution to the RNI as well as possible heavy metal exposure.

## 5. Conclusions

Sardines and anchovies were the most nutrient rich species sampled in this investigation. Further, we found that whole fish were substantially more nutrient dense than fillet samples of sardines and anchovies, mainly with regard to vitamin A, iodine, zinc, calcium, and iron. However, horse mackerel and axillary seabream, even though only fillet samples were analyzed, were also nutrient rich and could be considered to be important dietary sources of especially vitamin D, B_12_, EPA, DHA, and iodine. Iodine and selenium are considered important nutrients which are highly variable and often driven by a wide array of abiotic and biotic factors [17], and therefore these new analytical data may be useful in order to provide local values for these nutrients.

## Figures and Tables

**Table 1:** Overview of species sampled, type of sample, and number of fish in each sample from Northwestern African marine ecosystems, May 2017.

Common Name	Scientific Name	Tissue Sampled	Type of Sample	Number of Sampling Positions	Number of Individual Samples	Number of Composite Samples	Number of Specimens in Composite Sample
European pilchard	*Sardina pilchardus*	Whole fish	Composite	3	0	9	25
Eurpean pilchard	*Sardina pilchardus*	Fillet with skin and bones	Composite	3	0	9	25
Anchovy	*Engraulis encrasicolus*	Whole fish	Composite	1	0	3	25
Anchovy	*Engraulis encrasicolus*	Fillet with skin and bones	Composite	2	0	6	25/15 ^b^
Atlantic horse mackerel	*Trachurus trachurus*	Fillet	Individual/Composite ^a^	2	25	3	5
Axillary seabream	*Pagellus acarne*	Fillet	Individual/ Composite ^a^	1	50	6	5

**^a^** For Atlantic horse mackerel and axillary seabream, the individual fish samples were pooled to a composite sample for the vitamin analyses. For the rest of the analytes, individual fish were analyzed. **^b^** Three composite samples with 15 fish and three with 25 fish.

**Table 2:** Species, habitat, weight, and length of fish sampled on the EAF Dr. Fridtjof Nansen cruise from Tangier to Cap Bojador in May 2017.

Common Name	Scientific Name	Local Name (FAO, 1998)	Habitat	Weight, g ^a^	Length, cm ^a^
Sardine ^b^	*Sardina pilchardus*	Sardine	Pelagic	52 (34–73)	17 (16–20)
Anchovy ^b^	*Engraulis encrasicolus*	Anchois, Cheton, Lanchouba	Pelagic	13 (11–19)	13 (12–14)
Atlantic horse mackerel	*Trachurus trachurus*	Chinchard, Chrenne, Hringa, Saurel, Jurel	Pelagic	323 (180–390)	31 (26–35)
Axillary seabream	*Pagellus acarne*	Pageot, Besugo, Bokha, Boka, Boubrahim, Taznaght	Pelagic	294 (160–510)	25 (21–29)

^a^ Values given as mean (min-max). ^b^ Weight and length are given as mean of all the composite sample means.

**Table 3:** Analytical values for energy, total fat, and total protein in different species of fish sampled on the EAF Dr. Fridtjof Nansen cruise from Tangier to Cap Bojador in May 2017 (mean ± SD) ^a^.

Seafood		KJ/100 g	Kcal/100 g	Water g/100 g	Ash g/100 g	Total Fat g/100 g	Total Protein g/100 g
Common Name	Sample						
Sardine	Whole fish (*n* = 9) ^b^	673 ± 115	161 ± 27	70.7 ± 3.0	3.6 ± 0.33	7.8 ± 3.5	17.6 ± 1.1
Sardine	Fillet with skin (*n* = 9) ^c^	688 ± 87	164 ± 21	71.7 ± 2.4	2.4 ± 0.14	7.5 ± 2.4	19.3 ± 1.1
Anchovy	Whole fish (*n* = 3) ^d^	590 ± 28	141 ± 7	73.6 ± 1.5	3.0 ± 0.31	4.3 ± 0.4	18.7 ± 0.9
Anchovy	Fillet with skin (*n* = 6) ^e^	552 ± 50	132 ± 12	75.4 ± 1.8	1.9 ± 0.19	4.2 ± 1.1	19.2 ± 0.7
Atlantic horse mackerel	Fillet (*n* = 25)	401 ± 21	96 ± 5	80.7 ± 1.7	1.4 ± 0.08	0.61 ± 0.28	17.3 ± 1.6
Axillary seabream	Fillet (*n* = 50)	482 ± 19	115 ± 5	78.0 ± 1.6	1.5 ± 0.06	0.93 ± 1.1	19.2 ± 0.9

^a^ Values are given in mean ± SD. ^b^ Nine composite samples were analyzed consisting of in total 225 fish. ^c^ Nine composite samples were analyzed consisting of in total 225 fish. ^d^ Three composite samples were analyzed consisting of in total 75 fish. ^e^ Six composite samples were analyzed, consisting of in total 120 fish.

**Table 4:** Analytical values for total fat and fatty acids in different species of fish sampled on the EAF Dr. Fridtjof Nansen cruise from Tangier to Cap Bojador in May 2017 (mean ± SD) ^a^.

Seafood		Sum SFA g/100 g (%) ^b^	Sum MUFA g/100 g (%) ^b^	Sum PUFA g/100 g (%) ^b^	Sum *n*-3 g/100 g (%) ^b^	Sum *n*-6 g/100 g (%) ^b^	EPA g/100 g (%) ^b^	DHA g/100 g (%) ^b^
Common Name	Sample							
Sardine	Whole fish (*n* = 9) ^c^	1.9 ± 0.64 (31)	1.2 ± 0.52 (18)	2.9 ± 1.1 (45)	2.6 ± 1.1 (41)	0.20 ± 0.04 (3)	1.2 ± 0.82 (17)	0.87 ± 0.15 (16)
Sardine	Fillet with skin (*n* = 9) ^d^	2.0 ± 0.69 (31)	1.2 ± 0.52 (18)	3.1 ± 1.3 (46)	2.8 ± 1.1 (42)	0.02 ± 0.04 (3)	1.3 ± 0.91 (17)	0.93 ± 0.15 (16)
Anchovy	Whole fish (*n* = 3) ^e^	1.2 ± 0.01 (31)	0.60 ± 0.01 (15)	1.9 ± 0.03 (48)	1.8 ± 0.03 (44)	0.14 ± 0.004 (4)	0.54 ± 0.01 (14)	1.0 ± 0.02 (25)
Anchovy	Fillet with skin (*n* = 6) ^f^	1.0 ± 0.32 (30)	0.60 ± 0.26 (17)	1.6 ± 0.39 (47)	1.5 ± 0.34 (43)	0.13 ± 0.05 (4)	0.41 ± 0.01 (13)	0.84 ± 0.21 (25)
Atlantic horse mackerel	Fillet (*n* = 25)	0.19 ± 0.09 (29)	0.12 ± 0.07 (17)	0.31 ± 0.10 (50)	0.27 ± 0.09 (44)	0.03 ± 0.01 (6)	0.06 ± 0.03 (9)	0.19 ± 0.05 (31)
Axillary seabream	Fillet (*n* = 50)	0.31 ± 0.34 (30)	0.23 ± 0.40 (18)	0.45 ± 0.32 (48)	0.39 ± 0.27 (42)	0.06± 0.05 (6)	0.06 ± 0.06 (6)	0.28 ± 0.15 (31)

^a^ Values are given in mean ± SD. ^b^ Percent of total fatty acids. ^c^ Nine composite samples were analyzed consisting of in total 225 fish. ^d^ Nine composite samples were analyzed consisting of in total 225 fish. ^e^ Three composite samples were analyzed consisting of in total 75 fish. ^f^ Six composite samples were analyzed, consisting of in total 120 fish. SFA: saturated fatty acids; MUFA: mono-unsaturated fatty acids; PUFA: poly-unsaturated fatty acids; EPA: eicosapentaenoic acid; DHA: docosahexaenoic acid.

**Table 5:** Analytical values for selected amino acids in different species of fish sampled on the EAF Dr. Fridtjof Nansen cruise from Tangier to Cap Bojador in May 2017 (mean ± SD) ^a^.

Seafood		Amino Acids									
Common Name	Sample	Valine g/100 g	Leucine g/100 g	Isoleucine g/100 g	Phenyl- Alanine g/100 g	Histidine g/100 g	Metionine g/100 g	Threonine g/100 g	Lysine g/100 g	Trypto- Phan g/100 g	Taurine g/100 g
Sardine	Whole fish (*n* = 9) ^b^	0.79 ± 0.07	1.1 ± 0.10	0.63 ± 0.06	0.62 ± 0.05	0.61 ± 0.06	0.45 ± 0.03	0.67 ± 0.05	1.4 ± 0.14	0.19 ± 0.04	0.29 ± 0.01
Sardine	Fillet with skin (*n* = 9) ^c^	0.88 ± 0.08	1.3 ± 0.11	0.73 ± 0.06	0.72 ± 0.06	0.82 ± 0.08	0.51 ± 0.04	0.75 ± 0.06	1.6 ± 0.14	0.20 ± 0.01	0.17 ± 0.01
Anchovy	Whole fish (*n* = 3) ^d^	0.87 ± 0.05	1.3 ± 0.08	0.69 ± 0.04	0.72 ± 0.05	0.78 ± 0.05	0.50 ± 0.03	0.73 ± 0.04	1.4 ± 0.10	0.2 ± 0.02	0.26 ± 0.02
Anchovy	Fillet with skin (*n* = 6) ^e^	0.93 ± 0.07	1.4 ± 0.08	0.77 ± 0.06	0.76 ± 0.04	0.98 ± 0.08	0.53 ± 0.03	0.76 ± 0.04	1.6 ± 0.10	0.2 ± 0.01	0.18 ± 0.01
Atl. horse mackerel	Fillet (*n* = 25)	0.85 ± 0.09	1.4 ± 0.14	0.75 ± 0.08	0.71 ± 0.07	0.47 ± 0.09	0.55 ± 0.05	0.75 ± 0.08	1.7 ± 0.2	0.17 ± 0.02	0.15 ± 0.02
Axillary seabream	Fillet with skin (*n* = 50)	0.90 ± 0.05	1.5 ± 0.10	0.82 ± 0.06	0.81 ± 0.07	0.66 ± 0.09	0.58 ± 0.04	0.84 ± 0.06	1.8 ± 0.11	0.2 ± 0.02	0.12 ± 0.01

^a^ Values are given in mean ± SD. ^b^ Nine composite samples were analyzed consisting of in total 275 fish. ^c^ Nine composite samples were analyzed consisting of in total 275 fish. ^d^ Three composite samples were analyzed consisting of in total 75 fish. ^e^ Six composite samples were analyzed, consisting of in total 120 fish.

**Table 6:** Analytical values for selected vitamins in different species of fish sampled on the EAF Dr. Fridtjof Nansen cruise from Tangier to Cap Bojador in May 2017 (mean ± SD) ^a^.

Product		Vitamin D_3_ µg/100 g	Vitamin A_1_ µg/100 g	Vitamin E (α-Tocopherol), α TE/100 g (µg/100 g)
Common Name	Sample			
Sardine ^b^	Whole fish (*n* = 9)	9 ± 2.2	115 ± 32.7	288 ± 74
Sardine ^b^	Filet (*n* = 9)	10 ± 2.9	5.4 ± 1.9	394 ± 140
Anchovy ^d^	Whole fish (*n* = 3)	2 ± 0.5	125 ± 30.2	421 ± 113
Anchovy ^d^	Filet (*n* = 6)	1 ± 0.2	7.0 ± 2.8	436 ± 121
Atlantic horse mackerel ^c^	Filet (*n* = 3)	28 ± 17.4	4.2 ± 1.3	115 ± 14
Axillary seabream ^c^	Filet (*n* = 6)	2 ± 0.5	6.0 ± 8.8	364 ± 109

^a^ All values are presented as mean ± SD. ^b^ Sardine were analyzed as nine composite samples for both whole fish and filet, where each composite sample consisted of 25 individuals. ^c^ Horse mackerel were analyzed as three composite samples and Axillary seabream as six composite samples, where each composite sample consisted of five individuals. One value was <LOQ for Axillary sea bream. ^d^ Anchovy were analyzed as three (whole fish) and six (fillet) composite samples, where each composite sample consisted of 25 individuals.

**Table 7:** Analytical values for the B-vitamins in different species of fish sampled on the EAF Dr. Fridtjof Nansen cruise from Tangier to Cap Bojador in May 2017 (mean ± SD) ^a^.

Product		Vitamin B_1_ Thiamin mg/100 g	Vitamin B_2_ Riboflavin mg/100 g	Vitamin B_3_ Niacin mg/100 g	Vitamin B_6_ Pyridoxine mg/100 g	Vitamin B_9_ Folic acid µg/100 g	Vitamin B_12_ Cobalamin µg/100 g
Common Name	Sample						
Sardine ^b^	Whole fish (*n* = 9)	0.02 ± 0.006	0.27 ± 0.074	6.3 ± 0.37	0.36 ± 0.026	39.4 ± 11.6	15 ± 1.2
Sardine ^b^	Fillet (*n* = 9)	0.01 ± 0.0007	0.33 ± 0.054	7.0 ± 0.6	0.48 ± 0.038	9.3 ± 4.3	13 ± 2.1
Anchovy ^d^	Whole fish (*n* = 3)	<0.01	0.21 ± 0.014	6.2 ± 0.23	0.49 ± 0.017	44.3 ± 6.5	14 ± 0.4
Anchovy ^d^	Fillet (*n* = 6)	0.01 ± 0.00082	0.19 ± 0.011	7.3 ± 0.39	0.61 ± 0.040	21.2 ± 4.8	11 ± 1.0
Atlantic horse mackerel ^c^	Fillet (*n* = 3)	0.1 ± 0.02	0.12 ± 0.0018	4.0 ± 0.36	0.23 ± 0.017	5.1 ± 0.1	4.0 ± 0.3
Axillary seabream ^c^	Fillet (*n* = 6)	0.05 ± 0.008	0.10 ± 0.0082	5.4 ± 0.46	0.27 ± 0.091	5.2 ± 0.4	4.5 ± 0.9

**^a^** All values are presented as mean ± SD. **^b^** Sardine were analyzed as nine composite samples for both whole fish and filet, where each composite sample consisted of 25 individuals. **^c^** Horse mackerel were analyzed as three composite samples and Axillary seabream as six composite samples, where each composite sample consisted of five individuals. **^d^** Anchovy were analyzed as three (whole fish) and six (fillet) composite samples, where each composite sample consisted of 25 individuals, two fillet samples and two whole fish samples were <LOQ for vitamin B1.

**Table 8:** Analytical values of selected minerals in in different species of fish sampled on the EAF Dr. Fridtjof Nansen cruise from Tangier to Cap Bojador in May 2017 (mean ± SD) ^a^.

Seafood		Iodine µg/100 g	Selenium µg/100 g	Calcium mg/100 g	Potassium mg/100 g	Magnesium mg/100 g	Zinc mg/100 g	Iron mg/100 g	Phosphorus mg/100 g	Sodium mg/100 g
Common Name	Sample									
Sardine ^b^	Whole fish (*n* = 9)	67.4 ± 7.8	71.7 ± 19.2	716 ± 200	391 ± 33	45.5 ± 3.1	2.0 ± 0.3	4.3 ± 0.8	579 ± 90	170 ± 19
Sardine ^b^	Fillet (*n* = 9)	27.9 ± 5.1	34.7 ± 7.7	309 ± 118	441 ± 34	32.6 ± 1.9	1.3 ± 0.2	1.2 ± 0.2	407 ± 46	62 ± 5.5
Anchovy ^c^	Whole fish (*n* = 3)	36.1 ± 2.7	38.2 ± 2.1	535 ± 56	416 ± 19	51.1 ± 2.6	2.2 ± 0.2	3.1 ± 0.2	553 ± 38	175 ± 9.1
Anchovy ^d^	Fillet (*n* = 6)	12.7 ± 4.1	23.5 ± 2.5	211 ± 34	424 ± 7.9	33.7 ± 1.4	1.3 ± 0.1	0.9 ± 0.1	361 ± 20	72 ± 8.0
Atlantic horse mackerel	Fillet (*n* = 25)	25.2 ± 7.7	30.1 ± 4.3	46 ± 28	443 ± 25	30.2 ± 2.3	0.3 ± 0.03	0.9 ± 0.1	251 ± 16	50 ± 13
Axillary seabream	Fillet (*n* = 50)	35.1 ± 12.5	48.4 ± 8.3	50 ± 37	482 ± 26	32.1 ± 1.5	0.4 ± 0.1	0.5 ± 0.1	283 ± 20	41 ± 4.5

^a^ Values are given in mean ± SD. ^b^ Nine composite samples were analyzed consisting of in total 275 fish. ^c^ Three composite samples were analyzed consisting of in total 75 fish. ^d^ Six composite samples were analyzed, consisting of in total 120 fish.
